# Dynamic Natural Killer Cell and T Cell Responses to Influenza Infection

**DOI:** 10.3389/fcimb.2020.00425

**Published:** 2020-08-18

**Authors:** Kayla Frank, Silke Paust

**Affiliations:** ^1^Department of Immunology and Microbiology, The Scripps Research Institute, La Jolla, CA, United States; ^2^The Skaggs Graduate Program in Chemical and Biological Sciences, The Scripps Research Institute, La Jolla, CA, United States

**Keywords:** influenza virus infection, innate immunity, natural killer cell, T cell, lung resident immune cells

## Abstract

Influenza viruses have perplexed scientists for over a hundred years. Yearly vaccines limit their spread, but they do not prevent all infections. Therapeutic treatments for those experiencing severe infection are limited; further advances are held back by insufficient understanding of the fundamental immune mechanisms responsible for immunopathology. NK cells and T cells are essential in host responses to influenza infection. They produce immunomodulatory cytokines and mediate the cytotoxic response to infection. An imbalance in NK and T cell responses can lead to two outcomes: excessive inflammation and tissue damage or insufficient anti-viral functions and uncontrolled infection. The main cause of death in influenza patients is the former, mediated by hyperinflammatory responses termed “cytokine storm.” NK cells and T cells contribute to cytokine storm, but they are also required for viral clearance. Many studies have attempted to distinguish protective and pathogenic components of the NK cell and T cell influenza response, but it has become clear that they are dynamic and integrated processes. This review will analyze how NK cell and T cell effector functions during influenza infection affect the host response and correlate with morbidity and mortality outcomes.

## Introduction

Influenza is a rapidly mutating RNA virus which causes yearly global epidemics characterized by about 1 billion infections and 290,000–650,000 deaths (Iuliano et al., 2018). The current circulating serotypes are influenza A H1N1, influenza A H3N2, and influenza B/Victoria. The host develops memory responses following natural infection, however antigenic shift—mutations within a serotype—and antigenic drift—recombination of two different serotypes—create new variants to which the host may be naïve. Influenza A, but not B, viruses undergo antigenic drift because they infect a variety of animals. A pandemic may occur when this produces a novel transmissible strain to which there is no pre-existing immunity. The most notable pandemic was in 1918 (Spanish Flu) and the most recent in 2009 (Swine Flu) (Van de Sandt et al., 2012). Both viruses were of the H1N1 serotype and caused more severe disease than seasonal infections. In addition to circulating and past pandemic strains, avian influenza A strains H5N1 and H7N9 are occasionally introduced into the human population. They cause severe disease in humans but are not efficiently spread from person to person. However, there is concern that accumulation of mutations within an avian influenza virus could increase its transmission efficiency and cause a serious outbreak or global pandemic (WHO, 2005).

Typically, a seasonal influenza infection in healthy individuals is mild. However, complications often occur in the very young or old and in those with chronic conditions such as asthma, diabetes, or heart disease. Deaths attributed to influenza virus infection are not usually due to the virus itself. Instead, severe inflammation induces acute lung injury (ALI) and subsequent development of acute respiratory distress syndrome (ARDS) leads to death (Short et al., 2014). Avian influenza virus infections cause ARDS at a relatively high frequency and have higher morbidity and mortality rates than seasonal influenza virus infections (WHO, 2005).

Many research efforts focus on the treatment and prevention of influenza infection through B cell- mediated humoral immune mechanisms: neutralizing antibodies or vaccines (Reviewed in Krammer, 2019; Nachbagauer and Palese, 2020). NK cell and T cell cellular immune responses are equally important. The role of NK cells is poorly understood despite extensive contributions to the anti-viral influenza response. T cells are the less-studied arm of the adaptive immune system although they are arguably more essential to viral clearance than B cells (Bender et al., 1992; Graham and Braciale, 1997). Furthermore, both can mediate heterosubtypic immunity and enhance host protection following exposure to an influenza subtype to which the host does not have neutralizing antibodies (Braciale, 1977; Jegaskanda et al., 2013a). We will discuss NK cells and T cells and the two mechanisms which characterize their response to influenza infection: cytokine production and cytolysis of infected cells.

Many groups have attempted to dissect the relative contributions of individual immune response components in viral clearance and lung damage. Inconsistencies in experimental design and analysis underlie a body of influenza-related literature offering conflicting conclusions. We will discuss existing literature on the multifaceted NK cell and T cell responses to influenza A virus infection—mechanisms of activation, cytokine production and cytolysis of infected cells—and analyze the contributions of their effector functions to controlling viral replication and mediating lung immunopathology. To conclude, we will discuss therapeutic strategies which target aspects of the T cell and NK cell response to influenza infection.

## NK Cells

### Development

NK cells develop from the common lymphoid progenitor (CLP) in the bone marrow (Lanier et al., 1986b; Kondo et al., 1997). Transcription factors T-bet and Eomesodermin (Eomes) regulate NK cell development: dysfunction in both results in severe NK cell defects (Townsend et al., 2004; Intlekofer et al., 2005). In normal development, NK cells undergo continuous changes in receptor expression, commit to the NK lineage, and enter circulation as immature NK cells (Reviewed in Yu et al., 2013). Unlike T and B cells, NK cells do not express a rearranged antigen receptor. Instead, they express a diverse array of germline encoded activation and inhibition receptors. The balance between signals from these receptors determines their activation state and effector function (Lanier, 2008).

### Functions

NK cells perform a variety of anti-viral functions in humans and mice which can be characterized by direct killing of target cells and production of immunomodulatory cytokines. NK cells can kill target cells through natural and antibody-dependent cytotoxicity (ADCC). Natural cytotoxicity refers to NK cell killing of target cells in the absence of inhibitory signals from self MHCI molecules (Kärre, 1985; Kärre et al., 1986). ADCC is induced through binding of the NK stimulatory Fcγ receptor CD16 to the Fc region of IgG bound to a target cell (Trinchieri et al., 1975; Perussia et al., 1984). During natural and antibody-dependent cytotoxic responses, activated NK cells mobilize and release cytotoxic granules containing perforin and granzymes which initiate apoptosis in the target cell (Kägi et al., 1994a; Voskoboinik et al., 2006).

NK cell inhibitory receptors of the killer cell immunoglobulin-like receptor (KIR) (human) and Ly49 receptor (mouse) families bind self MHC I molecules and inhibit cytotoxicity toward healthy self cells; malignant or infected cells which downregulate MHC I are susceptible to NK cell killing (Karlhofer et al., 1992; Wagtmann et al., 1995b). KIRs are immunoglobulin-like receptors whereas Ly49 receptors are C-type lectin type II family homodimer proteins linked by a disulfide bond (Yokoyama, 1993; Wagtmann et al., 1995a). Both human and murine NK cells also express CD94/NKG2A, which recognizes non-classical MHC molecules and closely resembles mouse Ly49 receptor structure (Phillips et al., 1996). Murine NK cells express GP49 which is structurally and functionally homologous to human KIRs (Wang et al., 1997).

NK cells express a variety of other MHC-independent inhibitory and stimulatory receptors which contribute to the cell's activation state. As evidenced by KIRs and Ly49s, NK cell receptor repertoires vary between mice and humans. Therefore, the functions of various phenotypically distinct populations identified in murine models of disease may not always be physiologically relevant to humans. This is a challenge in translating NK cell research findings in mice to humans and should be considered in our discussion of NK cell function in mice.

MHC-independent inhibitory receptors limit potential NK cell-mediated immunopathology but can be the target of immune evasion mechanisms by infected or malignant cells. For example, programmed death pathway receptor PD-1 and its ligands PD-L1 and PD-L2 are expressed on NK cells, protecting against hyperinflammatory responses during infection (Quatrini et al., 2018). NK cells are increasingly credited as important contributors to the improved outcomes of patients receiving checkpoint blockade therapies targeting these immunosuppressive mechanisms (Wiesmayr et al., 2012; Beldi-Ferchiou et al., 2016; Hsu et al., 2018). Similarly, surface receptors Tim3, TIGIT, and Siglec-7 and−9 inhibit NK cell cytotoxic function when bound by their ligands on potential target cells (Stanietsky et al., 2009; Ndhlovu et al., 2012; Jandus et al., 2014). Therapeutic blockades of Tim3 and TIGIT have also shown efficacy as anti-cancer therapeutics in mice (Xu et al., 2015; Zhang et al., 2018). NK cell inhibitory receptors regulate NK cell cytotoxic responses which are vital for control of infection; more thorough characterizations of these receptors may be beneficial in the development of targeted therapies to enhance NK cell anti-viral functions.

To kill a target cell, NK cells also require activation signals which differ depending on the responding cell, target cell, or surrounding environment. NKG2D is an activating receptor in murine and human NK cells which binds to MICA, a stress ligand upregulated on malignant or infected cells (Bauer et al., 1999). Activating natural cytotoxicity receptors (NCRs) such as human NKp46 (murine NCR1), NKp30 and NKp44 also recognize a variety of pathogen-derived and stress-induced ligands (Mandelboim et al., 2001; Vitenshtein et al., 2016). The NKG2C/CD94 complex functions as an activating receptor which recognizes the non-classical MHC molecule HLA-E (human) or Qa-1^b^ (mice) (Lanier et al., 1998; Vance et al., 1999). 2B4 also serves as a conserved NK activating receptor which binds to CD48 (Mathew et al., 1993; Brown et al., 1998). These receptors, among others, are fundamental in activating NK cell cytotoxic functions.

NK cells also kill infected cells through perforin and granzyme-independent mechanisms: Fas and TRAIL. Fas receptor expression is altered in infected, malignant or otherwise stressed cells, and NK cells express its ligand CD94 (FasL). Binding to Fas by FasL induces apoptosis in the target cell through a caspase-dependent pathway (Arase et al., 1995; Hao and Mak, 2009). Tumor necrosis factor-related apoptosis inducing ligand (TRAIL) is another ligand expressed on the NK cell surface which induces apoptosis in sensitive target cells expressing DR4 or DR5 (Zamai et al., 1998). These mechanisms complement cytotoxic killing mechanisms and expand the protective effects of NK cells.

In addition to direct killing of target cells, NK cells secrete a variety of pro- and anti-inflammatory cytokines which directly affect the nature of the immune response. IFN-γ is crucial in their control of intracellular pathogens (Dunn and North, 1991; Orange et al., 1995). NK cells are recruited to the lymph nodes during infection where their secretion of IFN-γ has two functions in T cell activation: promote Th1 differentiation and enhance DC antigen presentation. In the absence of IFN-γ, protective T cell responses are significantly impaired (Huang et al., 1993; Gerosa et al., 2002; Martín-Fontecha et al., 2004). TNF-α secreted by NK cells also contributes to activation of DCs and macrophages, further enhancing antigen presentation (Bancroft et al., 1989; Walzer et al., 2005).

Alternatively, NK cells secrete anti-inflammatory cytokine IL-10 during systemic inflammation (Mehrotra et al., 1998; Perona-Wright et al., 2009). NK cell derived IL-10 has been reported to regulate CD8 T cell responses and to be protective against excessive tissue damage in chronic infections such as murine cytomegalovirus (MCMV) and Hepatitis C Virus. However, this could contribute to persistent infection and inability to clear the virus (De Maria et al., 2007; Lee et al., 2009; Ali et al., 2019). NK cell cytokine production is essential for their control of viral infections, but the downstream effects of each cytokine varies significantly depending on the nature of the infection.

### Phenotype Affects Function

NK cells are often referred to in terms of phenotypically distinct subsets which exhibit different effector functions. NK cells in the mouse belong to one of four populations in order of maturity: CD11b^−^CD27^−^→CD11b^−^CD27^+^→CD11b^+^CD27^+^→CD11b^+^CD27^−^. CD11b^−^ NK cells are found in the bone marrow, lymph nodes and liver, whereas CD11b^+^ NK cells are dominant in the spleen, peripheral blood and lung. CD11b^+^ cells are defined by their efficient IFN-γ production and cytotoxicity in response to stimulus compared to CD11b^−^ NK cells. CD11b^+^CD27^+^ NK cells are considered the most functional subset. They show higher proliferative capacity, more IFN-γ production potential, and stronger cytotoxic function than the more mature CD11b^+^CD27^−^ subset (Kim et al., 2002; Chiossone et al., 2009).

In humans, CD56^bright^CD16^−^ NK cells are similar but not homologous to CD11b^−^ murine NK cells. They are immature, abundant in lymphoid tissues and efficient cytokine producers. Alternatively, mature CD56^dim^CD16^+^ NK cells are more abundant in the periphery and have higher cytotoxic activity (Lanier et al., 1986a). CD56^bright^ cells express high levels of KIRs, whereas CD56^dim^ cells lose KIR expression and gain CD94 expression. CD56^dim^CD16^+^CD57^+^ NK cells are considered terminally differentiated. They have lower proliferative capacity and are unresponsive to cytokine stimulation, however they are sensitive to stimulation through CD16 (Lopez-Vergès et al., 2010). CD57^+^ NK cells modulate inhibitory receptor expression: as they differentiate they lose expression of NKG2A and gain expression of KIRs, increasing their threshold for stimulation (Björkström et al., 2010).

During infection, the phenotype of NK cells changes to reflect their activation state and effector function. Besides cytokine production and expression of cytotoxic granules, activated cells can be identified by a variety of activation markers. For example, CD69 is a marker of general NK cell activation (Borrego et al., 1993). CD107a (LAMP1) is upregulated in actively degranulating NK cells (Alter et al., 2004). CD38 is a marker of activation during viral infection: it is expressed on NK cells when they encounter influenza infected cells and is correlated with CD107a expression and IFN-γ production (Le Gars et al., 2019). These markers are commonly used to describe NK cell activation and function in various infections or disease.

### Phenotype in Peripheral Tissues

NK cells in mice and humans are found in a variety of secondary lymphoid and non-lymphoid organs including lymph node, spleen, liver, skin, uterus, peripheral blood, and lung (King et al., 1991; Grégoire et al., 2007). The developmental stage, phenotype and function of NK cells varies by tissue. NK cells in the lymph nodes are mostly CD11b^−^ or CD56^bright^CD16^−^ whereas in the peripheral blood they are predominantly CD11b^+^ or CD56^dim^CD16^+^ (Ferlazzo et al., 2004; Chiossone et al., 2009). NK cell subsets can migrate from blood to tissues dependent on chemokine receptor expression. For example up to 50% of liver NK cells are tissue-resident and maintained in the liver by their expression of CXCR6 (Paust et al., 2010; Hudspeth et al., 2016). CXCR3^+^ and CCR5^+^ NK cells migrate to the lungs during influenza infection. This is correlated with increased expression of their ligands on lung epithelial cells (Carlin et al., 2018). Chemokine receptor expression is often used to determine tissue-specific residency. Other surface receptors—CD69, CD103, and CD49a—promote tissue retention and are used to describe tissue-resident NK cells in general (Shiow et al., 2006; Walzer et al., 2007; Björkström et al., 2016). Various circulating and tissue-resident NK cell populations may have unique and critical functions in host protection, but a variety of challenges have limited the understanding of trafficking and tissue-specific roles of NK cells (Reviewed in Ferlazzo and Carrega, 2012; Björkström et al., 2016).

### Role in the Lung

NK cells are a prominent lymphocyte population in human and mouse lungs. In a healthy human lung, NK cells comprise 10–20% of all lymphocytes, most of which are CD56^dim^CD16^+^. They are a highly differentiated population with a CD57^+^NKG2A^−^ phenotype and high KIR expression (Marquardt et al., 2017). Similarly, murine NK cells represent about 10% of lung lymphocytes (Grégoire et al., 2007). NK cells in mouse lungs are also dominated by a mature CD11b^+^CD27^−^ phenotype and show properties of terminally differentiated cells: higher levels of inhibitory receptor CD94 and lower levels of stimulatory receptors NKG2D and NKp46 compared to spleen and bone marrow NK cells (Hayakawa and Smyth, 2006; Wang et al., 2012). In the 1980s, human lung NK cells were described as “functionally impotent” due to unresponsiveness to stimulation; lung alveolar macrophages and epithelial cells had a suppressive effect on NK cell function (Robinson et al., 1984). Expression of inhibitory receptors on lung NK cells may contribute to their minimal responses to stimulus and serve as an important tolerance mechanism in the exposed lung microenvironment.

Recent analysis of human lung NK cells revealed a small but distinct subset of CD69^+^ NK cells characterized by a CD56^bright^CD16^−^ phenotype and higher functionality (Marquardt et al., 2017). Another study characterized this population more specifically as CD56^bright^CD49a^+^CD103^+^CD69^+^ lung-resident NK cells which upregulate CD107a, granzyme B and IFN-γ upon stimulation (Cooper et al., 2018). In mice there is evidence that NK cells accumulate in the lung and show increased function after infection, but it is unclear whether this is due to recruitment of blood NK cells, response of lung-resident NK cells, or some combination of the two (Stein-Streilein et al., 1988; Wang et al., 2012). Despite previous thinking that lung NK cells are not capable of significant cytotoxicity or cytokine production, more studies must be done to identify potential *in vivo* stimulatory factors which activate lung NK cells in respiratory infections.

## T Cells

### Development

T cells also develop from the common lymphoid progenitor (Kondo et al., 1997). Progenitor cells migrate from the bone marrow to the thymus where they commit to the T cell lineage (Miller, 1961; Ford et al., 1966). The T cell receptor (TCR)—a rearranged antigen receptor through which T cells recognize peptides presented on MHC of an infected cell—develops in the thymus. VDJ recombination, mediated by RAG1 and RAG2 enzymes, ensures a high diversity in TCR specificity (Reviewed in Schatz and Ji, 2011). Developing cells undergo positive selection ensuring functional TCR/MHC interactions and negative selection deleting self-reactive TCRs before committing to a single positive CD4 or CD8 lineage (Kisielow et al., 1988; Bill and Palmer, 1989).

### Function

During infection, viral antigens move through the lymphatic system to the lymph nodes where they are presented on MHC by antigen presenting cells (APCs). Naïve T cells also circulate through the lymphatics and are activated by APCs in the lymph nodes (Guermonprez et al., 2002; von Andrian and Mempel, 2003). CD4^+^ and CD8^+^ T cells recognize antigens presented on MHC II and I, respectively. Following initial proliferation and differentiation in the lymph node, effector T cells travel through the blood to the site of infection where they are activated to exert their effector function (Marelli-Berg et al., 2008). After a period of weeks, the effector T cell population contracts and a smaller memory T cell population in formed. Memory T cells can be tissue-resident or circulating and can respond immediately to control a second infection by the same pathogen (Reviewed in Seder and Ahmed, 2003; Chang et al., 2014).

CD4^+^ and CD8^+^ T cells are activated through similar mechanisms, but they play unique functional roles in infection. The CD4^+^ T cell response orchestrates both cell-mediated (Th1) and humoral (Th2) immunity in response to foreign pathogens. After initial activation, differentiation is driven by cytokine-dependent transcription factor expression (O'Shea and Paul, 2010). IFN-γ and IL-12 initiate Th1 responses characterized by T-bet expression and IL-2 and IFN-γ production. This induces a cellular response against intracellular pathogens characterized by enhanced CD8^+^ T cell cytotoxicity and development of memory CD8^+^ T cells (Mosmann et al., 1986). Notably, T-bet is a prevalent NK cell transcription factor and IL-2 is a potent NK cell activator; NK cell IFN-γ production in these conditions amplifies Th1 responses (Domzig et al., 1983; Townsend et al., 2004). GATA3 expression induces Th2 responses that produce IL-4, IL-5, and IL-15 and promote B cell antibody production and memory development (Mosmann et al., 1986). CD4 T cells can also differentiate into Tfh, Th17, and T regulatory cells (Tregs). Tfh cells are important costimulatory cells for B cell development (Reviewed in Vinuesa et al., 2005). Th17 cells are highly inflammatory cells regulated by Rorγt which produce IL-17, IL-22, and IL-27 and are associated with tissue homeostasis during infection (Park et al., 2005). Tregs are characterized by Foxp3 expression; they dampen the immune response and limit lung injury during influenza infection through secretion of TGF-β and IL-10 (Sakaguchi, 2000).

CD8^+^ T cells, or cytotoxic T cells, kill infected or “altered-self” cells (Zinkernagel and Doherty, 1974; Blanden et al., 1975). They release cytotoxic granules following recognition of a foreign antigen presented on MHC I. CD8^+^ T cells also express FasL and TRAIL through which they induce apoptosis in target cells (Kägi et al., 1994b; Jeremias et al., 1998). Viruses including herpesviruses, poxviruses, and adenoviruses evade CD8^+^ T cell immunity through downregulation of class I MHC molecules (Andersson et al., 1985; York et al., 1994; Byun et al., 2009). This would leave the virally infected cells susceptible to NK cell cytotoxicity, but they often employ other evasion mechanisms which affect expression of NK cell activating ligands on infected cells. For example, Kaposi's sarcoma-associated herpesvirus disrupts surface expression of ligands to NKG2D and NCR NKp80 while myxoma virus downregulates Fas (Coscoy and Ganem, 2000; Guerin et al., 2002; Thomas et al., 2008).

T cell cytotoxicity can be altered by inhibitory receptors or cytokines in the environment. T cell “exhaustion” is characterized by increased expression of inhibitory receptors and can prevent excessive inflammation but result in impaired viral clearance. T cell inhibitory receptors, similar to those discussed for NK cells, include PD-1, Lag3, Tim3, and TIGIT (Grosso et al., 2009; Yu et al., 2009; Jin et al., 2010). Treg cytokines IL-10 or TGF-β contribute to inhibitory receptor expression (Wherry, 2011). T cell dysfunction is primarily studied in cancer or chronic infections such a HIV, hepatitis B/C, and LCMV clone 13 (McLane et al., 2019). However, there is evidence of dysfunctional T cell activities due to inhibitory receptors in acute viral infections as well (Erickson et al., 2012). T cell expression of inhibitory receptors or immunosuppressive cytokines is implicated both in impairing viral clearance and protecting against tissue damage. For example, in influenza infection one study found enhanced Tim3 function improved survival outcomes whereas another found the opposite (Sharma et al., 2011; Cho et al., 2012). To understand T cell responses, it is important to acknowledge the balance between these features. Furthermore, the simultaneous regulation of both T cells and NK cell cytotoxicity through these mechanisms has not been addressed. It is possible that inhibitory receptors are necessary to limit pathological consequences of one response but impair the other cell type's control of the infection.

### Phenotype in Peripheral Tissues

Both naïve and memory T cells circulate through the lymphatics and vasculature until they encounter cognate antigen, but some memory T cells reside in the tissues and are termed tissue-resident memory T (T_RM_) cells. T_RM_ cells are generally identified by the expression of CD69 and CD103, but this method is not entirely accurate as CD69 can be transiently expressed on activated circulating cells and there are some T_RM_ cells which do not express one or both of these markers (Masopust and Soerens, 2019). Furthermore, CD103 is more commonly required for CD8^+^ T_RM_ cell maintenance in tissues than for CD4^+^ T cells (Casey et al., 2012). Chemokine receptor expression is also used to identify organ specific T_RM_ cells. An example of this in the lung is described in section Role in the Lung. Like tissue-resident NK cells, T_RM_ cells are poised to respond rapidly to infection and therefore are important for immune surveillance and host protection. The development and maintenance of T_RM_ cells is reviewed more thoroughly elsewhere (Masopust and Soerens, 2019).

### Role in the Lung

In naïve mice, over 90% of T cells in the lung are circulating in the vasculature. During influenza and other infections or inflammatory responses, T cells accumulate transiently in the lung parenchyma (Turner et al., 2014). A multitude of chemokine receptors, adhesion molecules and their ligands have been proposed as regulators of T cell homing to the lung. CXCL16 expression in the lung is important in CXCR6^+^ T cell homing; this is responsible for the recruitment and maintenance of T_RM_ cells in the airways following influenza infection in mice (Morgan et al., 2005; Wein et al., 2019). Murine T cells are imprinted with lung-homing receptor CCR4 upon activation by lung DCs and elicit protective immune responses against influenza (Mikhak et al., 2013). Alternatively, the expression of CCR4 ligands CCL17 and CCL22 correlate with harmful lung inflammation in WT and humanized mouse models of asthma (Perros et al., 2009; Zhang et al., 2017). CCR5 and CXCR6 were similarly implicated in CD8^+^ T cell recruitment and related lung pathology in COPD patients (Freeman et al., 2007). Chemotaxis of T cells into the lung could play an important role in both T cell-mediated protection and pathology during lung inflammation.

Following respiratory virus infections, the population of T cells which persist as resident memory cells in the lung are correlated with protection upon secondary exposure. Both CD4^+^ and CD8^+^ memory cells exist in the lung of recovered mice, but the population is skewed to favor CD8^+^ T cells (Hogan et al., 2001a,b). A study of influenza infection in mice demonstrated influenza-specific lung-resident memory T cells were CD69^+^, with CD4^+^ T_RM_ cells co-expressing CD11a and CD8^+^ T_RM_ cells co-expressing CD103. In humans, lung-resident T cells showed similar expression patterns following influenza infection (Turner et al., 2014). During murine infection with *Mycobacterium tuberculosis*, CD4^+^CD69^+^CXCR3^+^ T_RM_ cells increase in the lung parenchyma. These cells show similar features to previously described hypofunctional lung-resident NK cells: decreased IFN-γ production and increased PD-1 expression. However, they are associated with decreased bacterial load upon infection (Sakai et al., 2014). Stimulation during infection could overcome the expression of inhibitory receptors or they could have another uncharacterized protective role.

The lung has three functionally distinct compartments which are important to analyze separately: tissue, airways, and vasculature. Many studies characterize responses of the lung in general, but do not look further into cells in the airways or vasculature. This potential limitation should be considered when interpreting data of lung immune cells. To be more thorough, the airways can be studied through bronchoalveolar lavage and cells circulating in the vasculature can be distinguished from cells resident in the tissue through intravascular staining (Anderson et al., 2012). Better understanding of the regulation and phenotypic characteristics of distinct lung immune cell populations will broaden knowledge of their protective or pathogenic roles during respiratory infections.

## Influenza Virus Infection

Influenza virus infects respiratory epithelial cells through HA binding of sialic acids on the cell surface. Infection normally occurs in the upper respiratory tract, but some infections—common in avian influenza—spread to the lower respiratory tract and lead to increased disease severity (Shinya et al., 2006; Van Riel et al., 2006). The lung epithelium employs a variety of mucosal immune mechanisms to combat infection. Infected epithelial cells and surrounding innate immune cells sense the presence of viral RNA through pattern recognition receptors and promote an antiviral state by producing interferons (IFNs), interferon stimulated genes (ISGs), and pro-inflammatory cytokines. The main cells which contribute to this initial response are tissue-resident macrophages and DCs. Cytokines and chemokines recruit circulating innate effector cells including neutrophils, monocytes, and NK cells (Iwasaki and Pillai, 2014; Chen et al., 2018). NK cells can directly kill infected cells while monocytes, alveolar macrophages, and neutrophils clear dead cells from the site of infection through phagocytosis (Hashimoto et al., 2007). During this time, B cells and T cells of the adaptive immune response are activated and migrate to the lung where they are essential for viral clearance. Innate immune responses must be able to efficiently activate this adaptive immune system to effectively clear the infection (Wells et al., 1979).

Initial contributions from both pro- and anti-inflammatory cytokines are important in preventing morbidity and mortality. In infected mice, gene expression peaks at 8 days post infection. Genes involved in immune activation, cell proliferation, and inflammatory anti-viral responses—type I and II interferons, TNF-α, chemokine receptors, and other pro-inflammatory cytokines—dominate the response (Pommerenke et al., 2012). In humans, the response is similar with increases in pro-inflammatory cytokines including IFN-γ, TNF-α, IL-17, and IL-6 and anti-inflammatory cytokine IL-10 (Hayden et al., 1998; Bermejo-Martin et al., 2009; Yu et al., 2011). Altered expression of particular cytokines can disrupt lung homeostasis and affect host outcomes. This is a major area of research with therapeutic potential. We will discuss cytokine responses in terms of NK cells and T cells specifically; the general immunomodulatory role of cytokines in influenza infection has been reviewed elsewhere (Liu et al., 2016; Guo and Thomas, 2017).

Controlling viral replication and limiting immune-mediated tissue damage in influenza infection requires delicate balance. The lung epithelium employs a variety of mechanisms to limit unnecessary tissue damage in healthy individuals, but many of these are downregulated during infection to allow for sufficient immune responses (Snelgrove et al., 2011). Numerous immune cells and signaling molecules have been implicated in both lung immunopathology and host protection during influenza infection. For example, macrophages and neutrophils are important in clearance of infected apoptotic cells, but excessive infiltration is linked to influenza-induced lung injury (Hashimoto et al., 2007; Perrone et al., 2008; Narasaraju et al., 2011). IL-1 prolongs survival of influenza-infected mice at the expense of lung immunopathology (Schmitz et al., 2005). IL-6 has protective effects in experimental models of influenza, but in humans high levels of IL-6 are associated with severe infection (Yu et al., 2011; Dienz et al., 2012). We will address similar discrepancies for NK cell and T cell specific factors. Studies often attribute conflicting conclusions to differences in viral strains, infection doses, or mouse genetic backgrounds. More work is needed to understand the biological relevance and therapeutic potential of this data.

## NK Cells and T Cells in Influenza Infection

### Recognizing Infected Cells and Activation

Although both NK cell and T cell responses to influenza are characterized by cytokine production and direct killing of infected cells, their mechanisms of activation vary. NK cells in the lung vasculature and tissue are the first to encounter influenza-infected cells. These NK cells vary in phenotype and function—some are immature, circulating cells whereas others are mature, lung-resident cells—and are important for initial viral control and recruitment of immune effector cells. Within 3 days following low dose influenza infection in mice, CXCR3^+^ and CCR5^+^ NK cells accumulate in the lung, airways, and lung-draining lymph nodes (Ge et al., 2012; Carlin et al., 2018).

Early studies of lung NK cells suggested natural cytotoxicity receptors (NCRs) NCR1 (mouse), NKp44 and NKp46 (human) encoded by the *Ncr1* gene bind to HA on the surface of infected cells. NCRs are traditionally viewed as stimulatory NK receptors. One study of influenza infection in *Ncr1*^−/−^ mice suggests they are necessary for protection (Arnon et al., 2001; Mandelboim et al., 2001; Gazit et al., 2006). However, the stimulatory potential of NCR1 and NKp46 binding to HA during influenza infection is debated in the field. One study challenged NKp46-dependent NK cell activation using a mouse model with a W32R substitution in *Ncr1*. The mutation disrupted NKp46 expression on the NK cell surface without deleting the whole gene. Lack of NKp46 expression was associated with increased *Helios*-dependent transcription factor expression and NK cell activation. The mutation resulted in higher levels of NK cell IFNγ production and significantly higher survival during influenza infection (Narni-Mancinelli et al., 2012). More work is needed to clarify conflicting data regarding NKp46/NCR1-dependent NK cell responses.

NK cell costimulatory receptors 2B4 and NTB-A bind to HA and enhance NK cell cytotoxicity, but cannot activate NK cells on their own (Duev-Cohen et al., 2016). Influenza virus has evolved immune evasion mechanisms for NK cell recognition of HA. For example, virally encoded NA glycoproteins remove sialic acid residues on NKp46/NCR1, disrupting human and murine NK cell recognition of HA (Bar-On et al., 2013). Alternatively, HA can internalize into NK cells and inhibit NKp44/46-induced cytotoxicity through disruption of the ζ signaling chain (Mao et al., 2010). Influenza can also directly infect mouse and human NK cells, removing potential cytotoxic cells from the environment (Guo et al., 2009; Mao et al., 2009). This could impair NK cell responses, but they possess alternative activation mechanisms.

CD16 (FCγRIII) binds to the Fc portion of influenza-specific antibodies and initiates ADCC. Studies in humans and macaques demonstrate seasonal influenza infections elicit cross-reactive antibodies to HA and NA epitopes of multiple influenza strains; these antibodies promote NK cell ADCC against infected target cells (Jegaskanda et al., 2013c, 2014). The presence of ADCC-promoting antibodies correlates with reduced disease severity in humans and could explain the trend during the 2009 H1N1 pandemic where older individuals—who were more likely to have cross-reactive antibodies in their serum—were not as affected as younger individuals (Jegaskanda et al., 2013b; Valkenburg et al., 2019). A study in immunocompromised HIV patients who had never received an influenza vaccine found they also had cross-reactive antibodies capable of mediating NK ADCC against pH1N1 and H3N2 strains, confirming these antibodies can emerge from natural exposure to seasonal influenza strains (Nehul et al., 2018). There is also evidence that humans produce antibodies to conserved internal proteins which activate NK cells *in vitro* (Jegaskanda et al., 2013a; Vanderven et al., 2016). Seasonal influenza infections promote the development of cross-reactive antibodies which promote NK cell ADCC during infection with various influenza strains. This is a physiologically relevant protection mechanism which should be considered in the development of influenza therapeutics and vaccines.

Stress-related mechanisms are also associated with changes in NK cell activation state. For example, NKG2D recognizes stress-induced ligands MICA/B and ULBP on infected macrophages and DCs, respectively (Sirén et al., 2004; Draghi et al., 2007). Additionally, ssRNA recognition through endosomal pattern recognition receptor TLR7 activates spleen and lung NK cells in response to influenza infection in mice (Stegemann-Koniszewski et al., 2018). Undoubtedly, there are more mechanisms through which influenza infection leads to NK cell activation and in order to fully understand the NK cell response we should work to understand mechanisms of activation beyond NCRs and CD16.

Over the last several years, extensive research established that adaptive-like NK cells exist, recognize viral antigens, and contribute to host protection from secondary infection with a variety of pathogens, including influenza. The details of NK memory responses are reviewed elsewhere (Cerwenka and Lanier, 2016; Paust et al., 2017). Briefly, memory NK cells localize to the liver due to expression of CXCR6 and mediate protective responses against secondary influenza infection in mice (Paust et al., 2010; Li et al., 2017). There is evidence that influenza vaccination in mice promotes development of memory NK cells which rapidly secrete large amounts of IFN-γ upon viral challenge (Dou et al., 2015; Goodier et al., 2016). The recognition mechanisms which mediate NK cell antigen-specific responses are unclear. Future studies which address this for influenza and other pathogens will help with efforts to target NK cell responses therapeutically.

Antigen-specific T cells recognize influenza-infected cells through TCR binding to virally encoded peptides presented on MHC. Naïve influenza-specific T cell clones are activated by DCs and undergo proliferation in the lymph nodes before they become functional effector cells (Lawrence and Braciale, 2004). CCR7^−/−^ mice with inefficient DC migration to the lymph node fail to mount a protective influenza-specific T cell response (Heer et al., 2008; Ho et al., 2011). Following initial activation and proliferation in the lymph nodes, T cells return to the lung where they are activated by infected cells (Román et al., 2002; Legge and Braciale, 2003).

T cells recognize a variety of external and internal influenza proteins. Conserved surface matrix protein 1 (M1) and intracellular nucleoprotein (NP) are important for T cell-mediated heterosubtypic immunity to influenza. Significant research in the 1970s and 80s identified T cell immunity to NP and M1 were responsible for cross-reactivity between strains whereas T cell clones specific for HA or NA only mediated strain-specific protection (Braciale, 1977; Townsend et al., 1984). Cross-reactive T cells were identified in recent years to novel influenza strains: pH1N1, H5N1, and H7N9. In each case, cross-reactive T cells from healthy patients were characterized *in vitro* and their presence was further correlated with protection against morbidity and mortality in humans and mice (Lee et al., 2008; Guo et al., 2011; Sridhar et al., 2013; McMaster et al., 2015). In one study following pH1N1 patients, the presence of IFN-γ^+^IL-2^−^ cross-reactive memory CD8^+^ T cells specifically was strongly correlated with less severe disease (Sridhar et al., 2013). One universal influenza vaccine approach involves enhancing T cell-meditated heterosubtypic immunity to conserved epitopes. Although the presence of cross-reactive T cells seems to be protective, studies should address cytokine production of these cells to understand the potential effects of enhancing cross-reactive T cell responses on T cell-mediated lung immunopathology.

### Effector Functions

**Figure 1:**
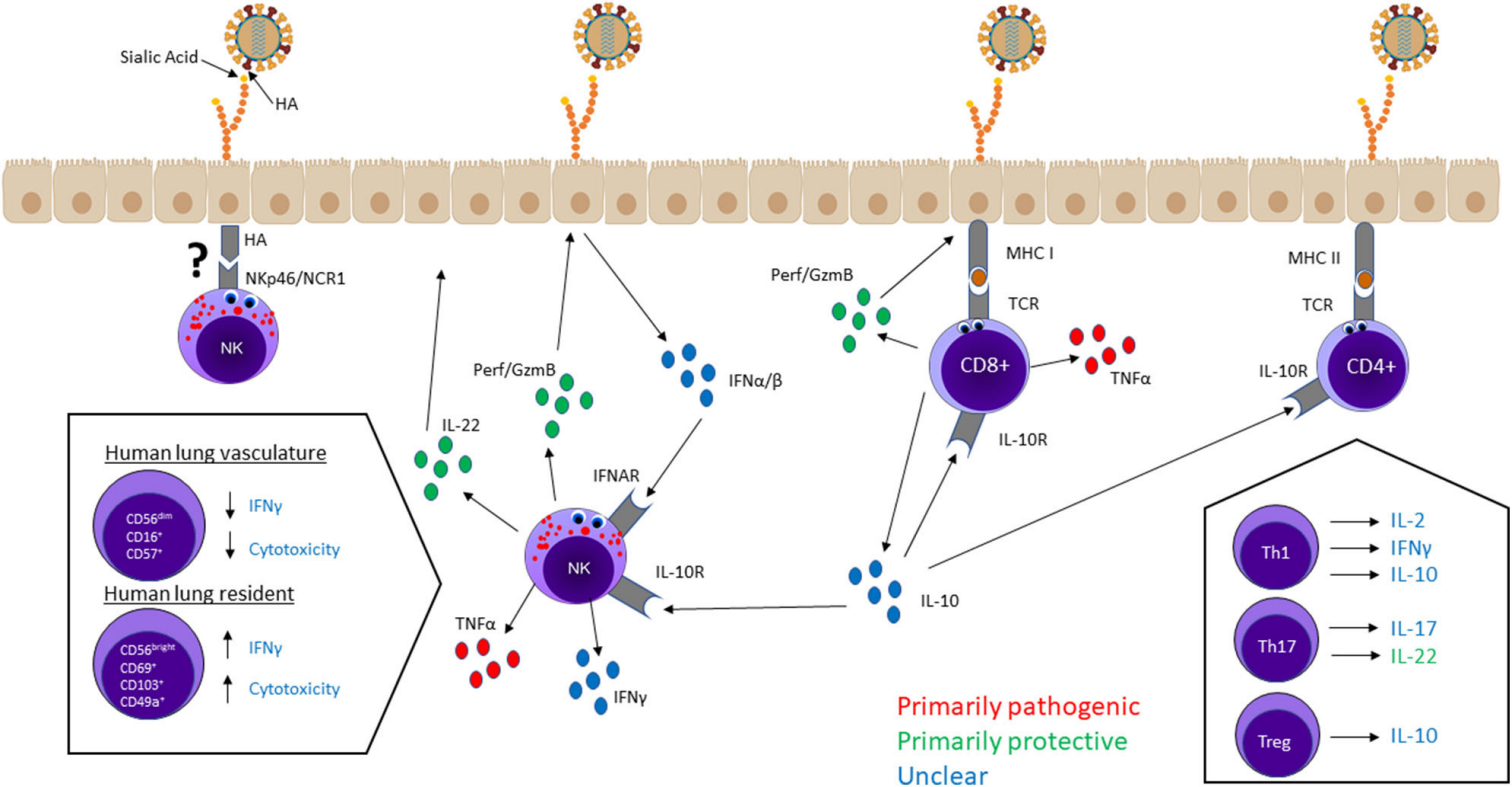
NK cell and T cell effector functions in the lung—cytokine secretion and cytotoxicity—mediate viral clearance and lung immunopathology. Most lung NK cells are terminally differentiated hypofunctional cells circulating in the lung vasculature, whereas a small population of immature lung-resident cells are capable of higher cytotoxicity and cytokine production. CD8^+^ T cells and NK cells mainly kill infected cells through cytotoxic granules containing perforin and granzymes. Cytotoxicity is not believed to have serious pathological consequences. T cells and NK cells produce a variety of cytokines, some of which—IFN-γ, IL-2, IL-10, IL-17—have unclear effects on host outcome. TNF-α seems to be a significant contributor to lung damage but not required for protection. Alternatively, tissue-regenerative cytokine IL-22 is considered an important protective factor. IL-10 regulates NK cell and T cell responses, but it is unclear whether higher levels of IL-10 improve or worsen host outcomes.

Unique functional populations of NK cells and T cells contribute significantly to influenza responses through cytokine production and/or direct killing of infected cells. In general, the cell phenotype, mechanism of activation, and surrounding cytokine milieu are determinants of each cell's effector function. Here, we analyze NK and T cell effector functions and their contributions to lung immunopathology during influenza infection. The main ideas from this section are visualized in Figure 1.

#### Cytokine Production

Phenotypically immature NK cells and CD4^+^ T cells are the predominant cytokine producing subsets in influenza infection. Among CD4^+^ T cells, the Th1 response dominates over the Th2 response. Evidence suggests an increased Th2 response or presence of Th2 cytokines impairs anti-influenza immunity; mice treated with IL-4 do not have efficient CD8^+^ T cell activation and clear influenza infection slower than untreated controls (Moran et al., 1996). Th17 and Treg contributions to the host response are characterized by unique cytokine production patterns: Th17 cells secrete IL-17 and IL-22 whereas Tregs secrete IL-10 and TGF-β. CD8^+^ T cells can secrete cytokines as well, although they are more often characterized as contributors to the cytotoxic response. Cytokines affect all aspects of the immune response: they can promote inflammation and prime immune responses, inhibit inflammation and prevent excessive tissue damage, or both.

IFN-γ is a pro-inflammatory cytokine produced by both NK cells and Th1 cells. Mice administered IFN-γ blocking antibodies have impaired humoral and cell-mediated immunity and significantly lower survival rates following influenza infection than IFN-γ-sufficient mice (Baumgarth and Kelso, 1996; Ishikawa et al., 2010). NK cell IFN-γ production in the lung is generally attributed to circulating cells. However, human lung-resident CD56^bright^CD49a^+^CD69^+^CD103^+^ NK cells are potent IFN-γ producers in response to influenza infection (Cooper et al., 2018). Th1 cells in the lungs and airways secrete high levels of IFN-γ. This contrasts Th1 cells in the lymph nodes which primarily produce IL-2 (Brown et al., 2004). Th1 derived IL-2 in the lymph node promotes CD8^+^ T cell secretion of IFN-γ during DC-dependent activation (Blachère et al., 2006). IFN-γ and IL-2 together promote cellular immune responses necessary for control of influenza infection: CD8^+^ T cell activation and B cell differentiation (Sarawar and Doherty, 1994).

In IFN-γ^−/−^ mice, DCs and T cells show decreased migration to the lymph nodes and limited influenza-specific responses in the lung. This is rescued by adoptive transfer of WT NK cells; NK cell-derived IFN-γ alone is sufficient for T cell activation during influenza infection, while T cell-derived IFN-γ is complementary (Ge et al., 2012). IFN-γ from NK cells also promotes antibody class switching to IgG2a and suppresses IgG1, IgG2b, IgG3, and IgE production (Snapper and Paul, 1987). NK cells contribute further to antibody production through direct contact with B cells: CD11a/CD54 (human), CD40L/CD40 (human), and CD2/CD48 (mouse) binding. One mechanism is described in mice where NK cells induce expression of germline transcripts which are necessary for IgG2a production (Gray and Horwitz, 1995; Blanca et al., 2001; Gao et al., 2005). It is efficient for IFN-γ to favor IgG2a production, as the NK cell ADCC receptor CD16 is an Fcγ receptor which preferentially binds the Fc region of IgG antibodies (Perussia et al., 1984). NK cell and B cell interactions are promoted by IFN-γ and are essential for antibody production and NK cell cytotoxicity, two vital host protective mechanisms against influenza infection.

Type I IFN signaling through IFN-α/β receptor (IFNAR) stimulates NK and Th1 IFN-γ production during influenza infection (Sareneva et al., 1998; Hwang et al., 2012). Multiple cells with lung-resident populations have been implicated as a source of type I IFN during influenza infection including monocytes, macrophages, DCs, and bronchial epithelial cells (Sirén et al., 2004; Draghi et al., 2007; Kronstad et al., 2018). IL-12, IL-18, and IL-2 have also been studied as stimulators of IFN-γ production. IL-12^−/−^ and IL-18^−/−^ mice do not show significant changes in NK cell IFN-γ, CD69 or CD107a expression, but *in vitro* experiments show that IL-12 and IL-18 efficiently activate IFN-γ expression in infected Th1 cells (Sareneva et al., 1998; Hwang et al., 2012). IL-2 derived from Th1 cells promotes significant IFN-γ production by human CD56^bright^ peripheral blood NK cells following *in vitro* influenza infection (He et al., 2004). Furthermore, influenza vaccination promotes IL-2-dependent IFN-γ secretion by memory-like human NK cells *in vitro* following secondary exposure (Goodier et al., 2016). Another human peripheral blood study with the 2009 H1N1 strain, however, found that neither IL-2 nor T cells are required for NK IFN-γ production (Kronstad et al., 2018). IFN-γ levels may be impacted by other factors besides stimulation: a study in humans found high expression of inhibitory receptors CD54 and CD112 in patients with low NK cell IFN-γ production. This is a potential immune evasion mechanism through which influenza viruses impair NK cell IFN-γ production (Kronstad et al., 2018). The stimulatory mechanisms and effects of IFN-γ production on influenza infection control are well-studied but still not fully understood.

TNF-α secreted by NK cells and T cells contributes significantly to the pro-inflammatory cytokine response during influenza. Influenza infection induces low expression of chemokines CCL2, CCL5, CXCL8, and CXCL10 on lung epithelial cells. In the presence of TNF-α this chemokine expression increases and promotes recruitment of immune effector cells. TNF-α also increases expression of pro-inflammatory pathway intermediates—IRF1, IRF7, RIG-I, and IKKε–and reduces viral replication in cultured human lung epithelial cells infected with influenza (Seo and Webster, 2002; Matikainen et al., 2006; Veckman et al., 2006). CD8^+^ T cells produce significant amounts of TNF-α; binding of inhibitory receptor NKG2A on T cells is important in limiting excessive production of TNF-α during influenza infection (Zhou et al., 2008). CD16^−^CXCR3^+^CD49a^+^ NK cells also secrete TNF-α (Scharenberg et al., 2019). These cells seem to fit the phenotypic profile of previously described CD56^bright^CD69^+^CD49a^+^CD103^+^ lung-resident NK cells (Cooper et al., 2018). TNF-α production by NK cells and CD8^+^ T cells broadly activates pro-inflammatory and migratory responses which contribute to influenza infection control.

Th17 cells induce a highly inflammatory environment through secretion of IL-17. NK cells have also been shown to produce IL-17 in response to infections, but this has not been described in influenza specifically (Passos et al., 2010). IL-17 is associated with expression of pro-inflammatory cytokines—IFN-γ, G-CSF, IL-6, IL-1B, and TNF-α—and neutrophil recruitment in mouse lungs (Crowe et al., 2009). In section Balance Between Viral Clearance and Immunopathology, we discuss increasing evidence that pro-inflammatory functions of IL-17 mediate lung damage in severe human influenza infections.

NK and T cell-derived anti-inflammatory cytokines contribute to tissue homeostasis. Tregs, typically characterized by their IL-10 secretion, inhibit the function of influenza-specific CD4^+^ and CD8^+^ T cells. In their presence, CD4^+^ and CD8^+^ T cells in the lung are less proliferative (Haeryfar et al., 2005; Bedoya et al., 2013). CD8^+^ T cells produce IL-10 in response to IFNAR signaling, IL-27, and CD4^+^ T cell-derived IL-2 during influenza infection in mice. Disturbing IFNAR signaling or CD4^+^ T cell-derived IL-2 disrupts CD8^+^ T cell production of IL-10 but not IFN-γ (Sun et al., 2011; Jiang et al., 2016). Type I IFNs, IL-27, and IL-2 contribute to stimulating NK cell and CD4 T cell antiviral function; induction of CD8^+^ T cell IL-10 production by these signaling molecules is equally significant and should be considered in studies which use the presence of these cytokines as a measure of immune activation or host resistance to influenza infection. Some reports claim NK cells only secrete IL-10 during systemic infections (Perona-Wright et al., 2009). This could explain why there is no evidence of IL-10 production during influenza's localized infection, but more investigation is needed.

Recent work challenged the idea that IL-10 is purely anti-inflammatory. The study uncovered a complex process where IFN-γ producing Th1 cells secrete IL-10 in the early stages of infection and counteract NA-mediated activation of latent TGF-β in flu-specific CD4^+^ T cells. In this case, IL-10 suppresses viral evasion mechanisms and enhances IFN-γ and TNF-α production. In later stages of infection when NA is not present, IL-10 exhibits immunosuppressive properties and protects the host from excessive tissue damage (Dutta et al., 2015). It is unclear if this applies to specifically Th1-derived IL-10 and if these IL-10 producing Th1 cells are always active during influenza infection. Regardless, this critical observation complicates IL-10's role in influenza infection.

IL-22 is a tissue-regenerative cytokine secreted by NK cells and Th17 cells in the lung, trachea and airways during influenza infection. IL-22R is upregulated in alveolar epithelial cells after influenza-induced injury, and IL-22 promotes recovery of those damaged cells (Orange and Biron, 1996). Th17 secretion of IL-22 is a self-regulatory mechanism with counteracts IL-17 and promotes tissue homeostasis. Similarly, studies show IL-22 from NK cells is important in minimizing lung damage caused by their own pro-inflammatory responses (Guo and Topham, 2010; Kumar et al., 2013).

Cytokine responses of NK cells and T cells are a focus of influenza research. The effects of some are well-characterized whereas others have only been described in a few studies. Regardless, the field lacks a complete understanding of the individual contributions of each cytokine to host protection and lung damage. We discuss this further in section Balance Between Viral Clearance and Immunopathology.

#### Cytotoxicity

Influenza infection also promotes cytotoxic functions of NK cells and T cells which correlate with viral clearance and positive survival outcomes. Mice with perforin deficiencies have significantly lower survival rates when infected with the same influenza dose as WT mice, and their LD_50_ dose is about 10 times lower than WT. NK cells and T cells from perf^−/−^ mice do not show functional cytotoxic killing of infected cells and although their cytokine and antibody responses increase significantly, perf^−/−^ mice are unable to efficiently clear the infection (Liu et al., 2003). NK and T cells also kill infected cells through Fas/FasL and TRAIL/TRAIL-DR interactions, complementing perforin and granzyme-mediated cytotoxicity to improve host resistance to infection (Topham et al., 1997; Ishikawa et al., 2005).

NK cell cytotoxicity is typically affiliated with mature CD56^dim^CD16^+^ cells, but there is conflicting evidence of the responsiveness of this population in the lung (Robinson et al., 1984; Marquardt et al., 2017; Cooper et al., 2018). A recent study challenged the general acceptance of hyporesponsive lung NK cells; they observed increased NK cell cytotoxicity in both CD56^dim^ and CD56^bright^ subsets from human blood and lung following influenza infection (Scharenberg et al., 2019). CD56^bright^CD49^+^CD69^+^CD103^+^ lung-resident cells are also capable of mounting strong cytotoxic responses (Cooper et al., 2018). Contributions of different populations to total NK cell cytotoxic function have not been fully described *in vivo*, but it is clear there are NK cells in the lung and blood which mediate cytotoxic killing of influenza infected cells and contribute to viral clearance.

T cell cytotoxicity is mostly attributed to CD8^+^ T cells, but Th1 cells also lyse influenza infected cells and contribute to viral clearance (Graham et al., 1993, 1994). CD8^+^ T cells which are activated and proliferate in the lymph node have the potential to kill infected cells upon migration to the lung. However, CD8^+^ T cells which experience additional stimulatory interactions with DCs in the lung have higher cytotoxic function. When DCs are absent from the mouse lung, impaired CD8^+^ T cell activation correlates with higher mortality and viral titers (McGill et al., 2008).

Environmental stimuli can affect the magnitude of the cytotoxic response. For example, IFN-γ enhances cytotoxic activity of both cell types. IFN-γ depletion partially impairs NK cytotoxicity, but completely suppresses T cell cytotoxicity (Ishikawa et al., 2010). IL-18^−/−^ mice show significant reduction in lung NK cell cytotoxicity and have 20% lower survival rates in response to influenza infection. These mice have no detectable changes in CD8 T cell cytotoxicity, so changes in survival are likely associated with defective NK cell responses (Liu et al., 2004). Both NK cells and T cells directly kill infected cells, but are not identically activated or regulated. This is beneficial for the host response, as one can compensate for the other in different physiological conditions.

### Balance Between Viral Clearance and Immunopathology

The extent to which NK cell and T cell responses are protective before they begin to become pathogenic is highly debated. We will attempt to dissect this in terms of influenza infection; other articles address all aspects of the immune response in mediating lung immunopathology during respiratory infections (Damjanovic et al., 2012; Newton et al., 2016; Tavares et al., 2017). Mechanistic studies in mice attempt to characterize contributions of NK cell and T cell responses to host protection and pathology. There are conflicting hypotheses as to the effect of different cell populations and soluble mediators on optimal host responses: efficient viral clearance while maintaining lung homeostasis. Studies of human infections attempt to connect hypotheses from mouse models to real patient outcomes, but there is no clear consensus as to which are the most beneficial or detrimental aspects of the host response to influenza. The ability to understand the host response lies in understanding how a host's overall health is affected by viral replication and immune defense mechanisms given external factors such as viral serotype, severity of infection, and stage of the immune response.

Mouse models of influenza infection have correlated NK and T cells with both protection and tissue damage. IL-15 knockout mice have defects in multiple cell types including CD8^+^ T cells, NK cells, NKT cells and intraepithelial lymphocytes and are protected from lethal dose of influenza infection. Their immune response is characterized by less myeloid cell infiltration, increased IL-10 expression and decreased levels of pro-inflammatory cytokines IL-6 and IL-12. One study of IL-15 knockout mice attributed improved outcomes to limited CD8^+^ T cell-mediated damage, whereas another claimed the absence of NK cells was protective (Nakamura et al., 2010; Abdul-Careem et al., 2012). Likely, both contribute in some way which should be analyzed further. Neither of these studies looked at doses which were not lethal in wildtype mice. The literature suggests NK cells are associated with pathology in high dose infection due to more NK cell recruitment to the lung; the same question has not been answered so clearly for T cells (Zhou et al., 2013; Carlin et al., 2018). This is worth investigating, as severity of lung damage is largely dose dependent.

In addition to the viral dose, many hypothesize that whether NK cells have a positive or negative effect on host immune responses to influenza is dependent on the mouse genetic background. IL-15 knockout mice do not show major strain-dependent responses; antibody depletion of NK cells resulted in slightly better protection in B6 mice than BALB/C, but the authors attribute this to a more complete NK depletion in B6 mice (Abdul-Careem et al., 2012). Another study compared several strains of mice receiving high-dose influenza infection. After NK depletion, they found that only 129 mice exhibited decreased ability to control viral replication. NK cells from 129 mice were characterized by increased IFN-γ expression compared to other strains, but no differences in CD107a (Abdul-Careem et al., 2012). This suggests the protective effects in these mice are due to higher IFN-γ expression whereas cytotoxic functions of NK cells do not affect the ability to control the infection.

The role of NK cells in severe disease should be considered from the perspective of human infections as well. In humans infected with pandemic H1N1, NK cell lymphopenia in the blood has been reported in association with severe disease. One patient with a complete absence of peripheral blood NK cells even had detectable viral RNA in the blood. In this context, the absence of NK cells is harmful for human outcomes and viral control. However, this data was obtained from blood samples, not lung or airway, so it is possible that decreased NK cells in the blood is due to increased migration to the lung (Denney et al., 2010; Fox et al., 2012). Despite significant efforts, there is still no consensus as to whether NK cell responses are protective or pathogenic in influenza infection.

The role of the influenza-specific CD8^+^ T cell response in lung immunopathology has been studied extensively. A fundamental study presented contradictory roles: CD8^+^ T cell-deficient mice have reduced lung damage at the expense of lower survival (Wells et al., 1981). Cell-intrinsic contributions by T cells can be analyzed through adoptive transfer of HA-specific CD8^+^ T cells into a mouse constitutively expressing HA on its respiratory epithelial cells. This model revealed CD8^+^ T cell responses alone can induce significant host damage: weight loss, pro-inflammatory cytokine accumulation, monocyte recruitment, and death (Enelow et al., 1998). A variety of regulatory mechanisms induce inhibitory receptor expression and are essential to prevent excessive CD8^+^ T cell responses to influenza infection (Zhou et al., 2008; Erickson et al., 2016). Nevertheless, T cell responses are necessary to clear infections and understanding the mechanisms through which they are responsible for immunopathology during natural infections is important for developing effective therapeutic interventions.

Tregs limit immunopathology but can suppress protective anti-viral responses in an antigen-specific manner. They accumulate in the mouse lungs, airways, and mediastinal lymph nodes before effector T cells, but after NK cells, in response to influenza infection (Betts et al., 2012). They directly inhibit CD4^+^ and CD8^+^ T cell proliferation and anti-viral responses (Haeryfar et al., 2005; Bedoya et al., 2013). Tregs also inhibit NK cell cytotoxicity when exposed to influenza antigens, although the interactions between NK cells and Tregs are not thoroughly characterized (Trzonkowski et al., 2004). There is evidence, but limited mechanistic understanding, of their effect on innate immune responses: transferring Tregs into Rag^−/−^ mice improves survival outcomes by preventing excessive myeloid cell infiltration in the lung (Antunes and Kassiotis, 2010). Tregs are fundamental in promoting recovery; inhibiting Treg function following viral clearance increases neutrophil infiltration, promotes pro-inflammatory cytokines in the airways, and impairs weight gain (Moser et al., 2014). Overall, Tregs seem to be more beneficial than harmful to the host response: preventing lung damage improves survival outcomes more than the increased cytotoxic responses in their absence.

Despite unclear contributions of NK and T cells overall, many efforts attempt to characterize their role in immunopathology based on cytokine production. Both cell types produce TNF-α following influenza infection. Inhibiting TNF-α signaling leads to increased survival outcomes and decreased lung damage following lethal dose influenza infection. Transcriptional analysis following TNF-α blockade revealed an overall decrease in pro-inflammatory cytokines and increase in CD4^+^ T cells and NK cells in the lung. These results implicate CD8^+^ T cell—not CD4^+^ T or NK cell—secretion of TNF-α and subsequent cytokine storm as a main mediator of lung damage during lethal influenza infections (Belisle et al., 2010; Shi et al., 2013). Other studies demonstrate CD8^+^ T cell-derived TNF-α contributes to lung pathology by promoting inflammation and inducing apoptosis in alveolar epithelial cells. However, it is not required for T cell-mediated viral clearance in mice (Liu et al., 1999; Xu et al., 2004). Notably, none of these studies used lower infectious doses so it is unclear if TNF-α mediates detrimental lung damage in all infections. In human studies of 2009 pH1N1 infection, TNF-α levels were higher in hospitalized patients than mild cases. Within severe cases, the patients in non-critical states showed higher expression of TNF-α than those with critical illnesses (Bermejo-Martin et al., 2009; Yu et al., 2011). TNF-α may be protective among patients who develop severe disease. This study did not address lung damage like those in mice, but it is possible that although TNF-α induces lung damage during infection, it is necessary for a fully functional host response.

IFN-γ production is a significant part of the NK cell and T cell response to influenza infection. It has been implicated as both helpful and harmful in influenza infection. Adoptive transfer of HA-specific IFN-γ deficient CD8^+^ T cells showed that IFN-γ is not essential for optimal protection but is important for recruitment of effector cells and control of hyperinflammatory responses (Wiley et al., 2001). Another study showed that IFN-γ has protective effects in mice with intact NK cell responses but not in NK deficient mice (Weiss et al., 2010). Most recently, IFN-γ was observed to suppress innate lymphoid cell function. IFN-γ deficiency protected mice challenged with a lethal dose of influenza H1N1 virus (Califano et al., 2018). Due to IFN-γ's various roles in infection, it is likely these studies yield different results due to distinct experimental conditions and procedures. The challenge, as with other questions of protection vs. pathology in influenza virus, is determining which information is physiologically relevant to humans. IFN-γ expression and correlation with patient outcomes has been analyzed in various studies. Like with TNF-α, levels of IFN-γ in hospitalized pandemic H1N1 patients are significantly higher than in mild cases. However, within hospitalized patients high levels of IFN-γ is associated with less severe disease (Bermejo-Martin et al., 2009; Yu et al., 2011). More work should be done to further analyze the effects of IFN-γ on patient outcomes, especially in relation to NK cells and the functionality of their IFN-γ production.

The role of IL-17 in human and murine influenza infection, like other inflammatory cytokines, is highly controversial. IL-17^−/−^ mice exhibit less weight loss, decreased lung pathology, and increased survival in response to influenza infection. This could be due to decreased neutrophil infiltration or cytokine production in the lung; IL-17^−/−^ mice have lower levels of IFN-γ, G-CSF, IL-6, IL-1B, and TNF-α compared to WT mice (Crowe et al., 2009). A mouse model of 2009 pH1N1 infection also showed that blocking IL-17 signaling enhances survival outcomes (Li et al., 2012). Alternatively, a study of IL-10 knockout mice observed increases in the Th17 response were protective against high-dose infection. The protection was attributed to IFN-γ production and cytotoxic activity of Th17 cells in addition to elevated IL-17 levels (McKinstry et al., 2009). Knowing IL-17 plays a dynamic role in infection outcomes, numerous studies address Th17 responses in humans and their correlation with disease outcome. Some studies observe higher levels of IL-17 in patients who effectively control the infection compared with patients who experience severe disease or death (Almansa et al., 2011). Other studies claim the presence of IL-17 is associated with more severe disease. One study of human pandemic H1N1 infection found, like TNF-α and IFN-γ, IL-17 expression was increased in patients with severe disease but within all hospitalized patients the presence of IL-17 was associated with higher survival (Bermejo-Martin et al., 2009). The overlap in expression characteristics of TNF-α, IFN-γ, and IL-17 in patients with severe influenza infection is an interesting observation. More studies should investigate potential redundancies in their responses and attempt to differentiate which are truly essential for the observed protection in severe disease.

IL-22 is generally studied for its protective effects during recovery from influenza infection. IL-22^−/−^ mice recover slower and have increased lung damage and lymphocyte infiltration in the lungs and airways (Pociask et al., 2013). While Th17 cells produce IL-22 in the lung in response to a variety of stimuli, their contribution in influenza infection is not as established as that of NK cells. One study found murine lung CD27^−^ NK cells produce IL-22 when stimulated with IL-23 but observed no significant changes in weight loss or survival. Following this, another group showed NK cell-derived IL-22 is protective against excessive weight loss by promoting regeneration of tracheal epithelial cells. Although the first NK cell study claimed no significant effects in the absence of IL-22 and the second saw the opposite, both sets of data show a similar trend of increased weight loss in IL-22 depleted mice. The lack of significance could be due to different methods of IL-22 depletion; The first study used an IL-22 blocking antibody whereas the second used IL-22 knockout mice (Guo and Topham, 2010; Kumar et al., 2013). Further investigation in mice revealed that in lethal infections, IL-22 does not have any impact on the outcome, but in sublethal infections it is important for limiting inflammation and initiating tissue repair (Ivanov et al., 2013). It is unclear whether IL-22 production by NK cells and T cells has a physiologically relevant protective role in human influenza infections.

IL-10 can be produced by NK cells and multiple T cell subsets, but the contributions of different IL-10 sources to the host response to influenza is unclear. Some studies show that IL-10 deficient mice have higher influenza survival rates. However, other studies claim deficiencies in IL-10 signaling can increase morbidity and mortality (McKinstry et al., 2009; Sun et al., 2009, 2010). These conflicting results could be due to stimulatory roles of IL-10 during early stages of influenza infection discussed in section Cytokine Production. They reported higher morbidity and mortality in mice with either IL-10 deficiency or overexpression. This suggests a homeostatic level of IL-10 which does not induce too strong of an initial inflammatory response—but is still strong enough to prevent excessive tissue damage —is ideal for host protection in influenza infection (Dutta et al., 2015). In humans, high levels of IL-10 have been associated with severe disease, however this is often attributed to IL-10 mediated immune suppression impairing the ability of the patient to control the infection (Yu et al., 2011). A potential role for IL-10 in harmful hyperinflammatory responses has not yet been studied in association with severe disease in humans. It is also unclear whether excessive IL-10 production is a byproduct of highly pathogenic infections or if increased IL-10 directly contributes to severe disease progression in humans. Surely, more work must be done to understand the role of IL-10 in lung homeostasis during murine and human influenza infections.

One study described IL-27 as an important regulator of previously described aspects of the T cell response to influenza. IL-27R^−/−^ mice had significantly worse outcomes in terms of viral load, lung pathology and survival; they concluded that IL-27 protects mice from severe pathology by inhibiting excessive CD4^+^ T cell IFN-γ, IL-17, and TNF-α production. They treated WT mice with recombinant IL-27 (rIL-27) following lethal dose influenza infection and found lower IL-17 levels and higher IL-10 levels correlated with increased protection without compromising viral clearance. Interestingly, they only saw these effects when they treated with rIL-27 at peak viral titer or later, compared with worse outcomes if they treated at early stages of infection. Likely, the early inflammatory response is necessary for favorable outcomes, but as adaptive immune cells accumulate in the lung increased inflammation mediates harmful tissue damage (Liu et al., 2014). This study ties together information discussed about positive and negative aspects of the T cell response and suggests that many of these functions are regulated by IL-27. Complementary studies in mice and humans are needed to fully understand the impact of IL-27 on the host response to influenza infection.

While studies in mice are limited in their physiological relevance to human infections, human studies also present challenges in drawing conclusions. Mechanistic studies of the human lung are logistically difficult and rare, but analysis of PBMCs of infected patients does not always represent the site of infection. Therefore, human host responses cannot be adequately compared to mice. Many human studies of hospitalized patients are biased toward young children or older adults. Although these populations represent the most accessible cases, they may not represent influenza responses from all demographics. Similarly, many studies focus on severe viral strains such as pandemic H1N1 or avian influenza infections rather than analyzing immune responses to more mild seasonal influenza cases. While our understanding of why some cases of influenza develop into severe life-threatening diseases has progressed, there is much to learn about individual immune responses and how they can be manipulated to affect patient outcomes.

Non-human primates (NHPs) are an alternative model which address some challenges associated with mouse and human studies. They have been successfully infected with numerous influenza strains including 1918 and 2009 pandemic H1N1 (Kobasa et al., 2007; Safronetz et al., 2011), H5N1 (Baskin et al., 2009), and H9N2 (Zhang et al., 2013). NHP immune responses and gene expression patterns following influenza infection closely mirror those of humans (Baskin et al., 2004). Therefore, they will be an essential tool to expand our knowledge of the mechanisms through which NK cells and T cells contribute to lung pathology in human-like infections.

## NK Cells and T Cells in Influenza Therapeutics

There are ongoing efforts to develop therapeutics which prevent acute lung injury (ALI) and acute respiratory distress syndrome (ARDS) during influenza infection. Developing these drugs is challenging as preventing the inflammatory response entirely would lead to uncontrolled viral replication. It is difficult to identify individual components of inflammatory responses which are directly responsible for immunopathology vs. those which are essential host responses or are simply byproducts of the inflammatory environment.

Current anti-viral therapeutics are limited to two approaches: neuraminidase and M2 ion channel inhibitors. Both aim to limit replication and spread of the virus: NA inhibitors target its enzymatic activity and M2 ion channel blockers prevent its proton channel function. These therapies have limited potential, however, because of side effects and the emergence of resistant strains (Gubareva et al., 2000; Leonov et al., 2011). Additionally, if the virus has sufficiently activated the innate immune system before the patient is treated, limiting viral spread does not prevent pro-inflammatory responses which have already begun. NA inhibitors, for example, are only useful if they are administered within the first 2–3 days of infection (Gubareva et al., 2000). In order to treat patients experiencing “cytokine storm,” therapeutics must work to limit harmful inflammatory immune responses without inhibiting viral clearance.

Targeting of individual cytokines has proven difficult; each contributes to several anti-viral response mechanisms and inhibiting their functions can lead to uncontrolled viral replication. Cytokines important in NK cell and T cell immunity such as IFN-γ and type I IFN have been studied as potential targets to limit pro-inflammatory cytokine responses, but there is no clear benefit to targeting them therapeutically. Alternatively, TNF-α blockade has shown therapeutic potential to limit harmful inflammatory responses to influenza without impairing viral control. TNF-α blocking antibodies are of particular interest; there are already approved drugs with this activity used to treat autoimmune disorders such as Crohn's disease and rheumatoid arthritis (Karampetsou et al., 2010). Therapeutic blockade of TNF-α signaling improves morbidity and mortality outcomes following lethal dose influenza infection in mice (Shi et al., 2013). A lethal dose in mice does not recapitulate all potential human disease courses, so human studies of TNF-α blockade are necessary to further assess its therapeutic potential. Other pro-inflammatory cytokines and chemokines—which are not prominent features of NK cell and T cell immunity but do have important functions in influenza infection—have been studied for their therapeutic potential (Reviewed in Ramos and Fernandez-Sesma, 2015).

There are also efforts to broadly modulate inflammatory responses through corticosteroid treatments. Original evidence in a small cohort of patients suggested corticosteroids can reduce morbidity and mortality in patients with ALI and ARDS (Tang et al., 2009). This led to studies over the past several years which provide conflicting results on the effects of corticosteroids on influenza patient outcomes. One review and meta-analysis of data from 30 human studies found that corticosteroid treatment was not beneficial and was correlated with increased mortality rates, however they acknowledged that the data quality was not sufficient for fully conclusive analysis and that more studies are needed to determine any conditions in which corticosteroids are beneficial for survival outcomes (Lansbury et al., 2019).

It may be enlightening to understand the effect of this treatment on NK cells and CD8^+^ T cells. There is no data addressing this for influenza, but NK cells isolated from asthmatic patients exhibit worse cytotoxic killing and cytokine production following corticosteroid treatment (Duvall et al., 2017). Liver NK cells from patients treated with certain corticosteroids also show decreased proliferation, cytotoxicity and cytokine production in hepatitis C virus infections (Ohira et al., 2017). As for T cells, one study showed memory CD8^+^ T cells from asthmatic patients upregulate BLT1—a surface receptor involved in T cell migration to the lung—following corticosteroid treatment and this increases inflammation and lung damage. However, CD8^+^ T cells in influenza infection may have different properties than memory T cells exposed to chronic inflammation (Ohnishi et al., 2008). Inflammation contributes significantly to activating NK cells and T cells. In the future, studies should test if the lymphocyte population possesses adequate cytotoxic function to clear the virus following anti-inflammatory treatments.

Although targeting inflammatory processes is at the forefront of attempts to develop influenza therapeutics, there are emerging cell-based therapies enhancing NK cell and CD8^+^ T cell cytotoxicity to infected cells. ADCC is a cytokine-independent NK cell killing mechanism which could avoid hyperinflammatory responses. Activating ADCC would be especially relevant in influenza where development of broadly neutralizing antibodies has been unsuccessful but non-neutralizing cross-reactive antibodies are readily available in human serum. Antibodies to both NP and M2e have shown protective effects in mice along with the ability to activate human NK cell ADCC mechanisms (Carragher et al., 2008; El Bakkouri et al., 2011; Simhadri et al., 2015; Vanderven et al., 2016). Treatment with NK ADCC cell-activating antibodies could complement anti-inflammatory therapeutics by promoting NK cell killing of infected cells independently of inflammation. However, a certain level of inflammation is necessary to recruit NK cells to the lung, so more work should be done to understand optimal methods for promoting NK cell recruitment and ADCC in the lung.

Activation of T cell-dependent immunity is a major focus in the influenza vaccine field, but there are also vaccine-independent T cell activation methods with potential to control influenza infections. Bispecific T cell engaging (BiTE®) antibody constructs can activate T cells to kill target cells independent of antigen-specificity. BiTE® antibody constructs are fusions of 2 single chain fragment variable (scFv) molecules: one binds to the antigen of interest and the other binds to CD3ε on cytotoxic T cells. These have been implemented in the cancer immunotherapy field, and M2e-specific BiTE® constructs show protective effects in mice infected with influenza (Huehls et al., 2015; Pendzialek et al., 2017). This treatment approach has not been studied further in mice and humans but presents a new perspective on activating T cells to target influenza-infected cells. As more work is done with M2e-specific antibodies, this may become a more well-studied therapeutic option.

Various T and NK cell therapies—adoptive transfer with chimeric antigen receptor (CAR), autologous or allogenic T and NK cells—are promising cancer treatments. CAR T and NK cells can be targeted to a specific antigen by combining an antigen-specific Fv to an intracellular signaling domain which promotes cytotoxicity upon antigen binding (Gross et al., 1989; Müller et al., 2008; Garfall et al., 2015). Autologous transfer of *ex-vivo* activated tumor-specific T or NK cells derived from the patient can enhance cytotoxic activity (Spanholtz et al., 2010; Rosenberg et al., 2011). NK cells are unique in that allogenic, MHC-mismatched NK cells do not promote graft-vs. host disease. In fact, allogenic NK cells are a powerful tool because they are not inhibited by the host MHC molecules and therefore have a lower threshold for stimulation when they encounter a malignant cell (Lundqvist et al., 2006; Iliopoulou et al., 2010). Although these approaches are currently focused on cancer, they could be used to mediate more efficient viral clearance in high-risk patients infected with influenza.

Current therapies targeting NK cell and T cell responses are limited. Anti-inflammatory approaches are more commonly studied because hyperinflammatory responses leading to cytokine storm are a common cause of morbidity and mortality in severe influenza infections. These efforts are hindered by incomplete understanding of the mechanisms behind protective and pathogenic inflammatory responses. Furthermore, the contributions of cytotoxic NK and T cells to the inflammatory response and the effects of anti-inflammatory therapeutics on their functions are poorly understood. Some studies have moved away from efforts to target a single anti-viral mechanism; cell-based immunotherapy approaches could supplement anti-inflammatory therapeutics and promote more efficient viral clearance by cytotoxic NK and T cells. Preliminary data shows that NK cells and T cells can be activated to potently kill influenza-infected cells without contributing to damaging inflammatory responses, but their potential in humans has not yet been evaluated.

## Discussion

Despite 2018 marking 100 years since the deadliest influenza pandemic in history, our understanding of influenza virus infections is far from complete. Countless host protection mechanisms have been characterized in mice with much focus on a hallmark of severe disease: hyperinflammatory responses and subsequent cytokine storm. Numerous other studies have attempted to correlate human disease outcomes with these immune mechanisms. NK and T cells are at the forefront of these responses: cytotoxic killing of infected cells is essential for host survival, but NK cell and T cell pro-inflammatory functions may contribute to immunopathology. Through years of research, the field is still unable to clearly distinguish protective responses from harmful ones; Studies addressing this issue will be fundamental to develop effective treatments for severe infections.

Some therapeutic approaches attempt to limit inflammation, but until we can distinguish harmful inflammatory mechanisms from necessary anti-viral responses this will be difficult. Others are turning to cell-based therapies to activate NK cell and T cell cytotoxicity against infected cells, but support for these approaches is backed by limited data. Developing effective treatments for severe influenza infections will likely involve a combination of these strategies. Regardless, understanding NK and T cell responses—both cytotoxicity and cytokine production—will undoubtedly be vital to develop effective therapeutics which can improve disease outcomes for patients experiencing severe influenza infections.

## Author Contributions

KF and SP conceptualized the content of and wrote the article. KF generated the figure. All authors contributed to the article and approved the submitted version.

## Conflict of Interest

The authors declare that the research was conducted in the absence of any commercial or financial relationships that could be construed as a potential conflict of interest.
